# A Case of Fatal Chloroform Narcosis

**Published:** 1884-10

**Authors:** 


### A CASE OF FATAL CHLOROFORM NARCOSIS.

We desire to record the following interesting case of fatal chloroform narcosis:

On Wednesday, August sixth, three o'clock P. M., a laboring man, twenty-eight years old, presented himself at the office of Dr. D. A. K. Steele, southeast corner of State and Eighteenth streets, to have a recent wound dressed. The middle and ring fingers of the right hand had been mangled and partially torn off by machinery in a planing-mill. Dr. Steele advised amputation through the second phalanges. As the patient was suffering from pain and shock, and was excessively nervous, it was determined to administer an anæsthetic. Dr. C. C. Beery was requested to administer Squibb's chloroform. The man was lying flat on his back on an operating table, within three feet of an open window. The anæsthetic was exhibited by means of a loosely folded napkin, and the tension of the vapor was lowered by free admixture of air. At the expiration of ten or twelve minutes, anæsthesia was complete. Between two and one-half and three drachms of chloroform were administered. The stage of excitement was marked, and the patient struggled violently; the pulse was rapid and compressible. When unconsciousness was fully established, Dr. Steele picked up the hand to begin the operation. Suddenly a long, stertorous respiration was heard, the heart ceased to beat, the man became pallid, and breathing stopped. The pallor yielded to a dark, venous congestion, which gradually faded away.

Vigorous efforts at resuscitation were at once instituted. The tongue was drawn forwards by a pair of Volsella forceps, and artificial respiration attempted. The heart muscle was directly irritated by a fine aspirating needle. All efforts were of no avail. Death had resulted from cardiac paralysis.

The autopsy, forty-eight hours later, revealed the brain slightly congested; lungs markedly congested, and slightly œdematous; heart of normal size, with large deposit of fat upon and around the auricles, fatty infiltration of muscular fibers, wall thin and pale, valves very thin and elongated, atheromatous degeneration of coronary arteries and aorta; aorta slightly contracted; kidneys, liver and spleen congested; abdominal cavity contained about three ounces of bloody serum; the small intestines were hyperæmic.

The man presented a preternaturally old appearance, seeming at least ten years older than he really was. An examination of the heart, prior to the administration of the chloroform, revealed no evidence of organic disease. The heart was simply acting rapidly and feebly. No history of previous condition, with reference to rheumatism, drunkenness or other predisposing cause, was elicited. Of course the coroner's verdict attached no blame to the physicians.

Dr. Steele deserves, and will receive, the cordial sympathy of the profession.

Ether is the universally recognized anæsthetic in the United States, but there are many conditions which contra-indicate its use. The so-called "English mixture" of one hundred parts chloroform, thirty parts sulphuric ether, and thirty parts absolute alcohol, has won a well-deserved reputation in the United Kingdom and Continental Europe as a safer anæsthetic than pure chloroform. The "English mixture" is, nevertheless, dangerous, and demands care and experience in exhibition. Four deaths, undoubtedly due to this agent, have been reported within the recent past, from Billroth's Clinic.

